# Safety and Efficacy of Sodium Hyaluronate Gel and Chitosan in Preventing Postoperative Peristomal Adhesions After Defunctioning Enterostomy: A Prospective Randomized Controlled Trials

**DOI:** 10.1097/MD.0000000000002354

**Published:** 2015-12-28

**Authors:** Jiancong Hu, Dejun Fan, Xutao Lin, Xianrui Wu, Xiaosheng He, Xiaowen He, Xiaojian Wu, Ping Lan

**Affiliations:** From the Department of Colorectal Surgery (JH, DF, XL, XW, XH, XH, XW, PL); the Department of Digestive Endoscopy (DF, XL); Guangdong Provincial Key Laboratory of Colorectal and Pelvic Floor Diseases, the Sixth Affiliated Hospital, Sun Yat-sen University (JH, DF, XL, XW, XH, XH, XW, PL); and Guangdong Institute of Gastroenterology, Guangzhou, Guangdong, China (JH, DF, XL, XW, XH, XH, XW, PL).

## Abstract

Peristomal adhesions complicate closure of defunctioning enterostomy. The efficacy and safety of sodium hyaluronate gel and chitosan in preventing postoperative adhesion have not been extensively studied. This study aims to evaluate the safety and efficacy of sodium hyaluronate gel and chitosan in the prevention of postoperative peristomal adhesions.

This was a prospective randomized controlled study. One hundred and fourteen patients undergoing defunctioning enterostomy were enrolled. Patients were randomly assigned to receive sodium hyaluronate gel (SHG group) or chitosan (CH group) or no antiadhesion treatment (CON group) during defunctioning enterostomy. The safety outcomes included toxicities, stoma-related complications, and short-term and long-term postoperative complications. Eighty-seven (76.3%) of the 114 patients received closure of enterostomy, during which occurrence and severity of intra-abdominal adhesions were visually assessed by a blinded assessor.

Incidence of adhesion appears to be lower in patients received sodium hyaluronate gel or chitosan but differences did not reach a significant level (SHG group vs CH group vs CON group: 62.1% vs 62.1% vs 82.8%, *P* = 0.15). Compared with the CON group, severity of postoperative adhesion was significantly decreased in the SHG and CH group (SHG group vs CH group vs CON group: 31.0% vs 27.6% vs 62.1%; *P* = 0.01). There was no significant difference in the occurrence of postoperative complications and other safety outcomes among the 3 groups.

Sodium hyaluronate gel or chitosan smeared around the limbs of a defunctioning enterostomy was safe and effective in the prevention of postoperative peristomal adhesions.

## INTRODUCTION

Intra-abdominal adhesions are a common complication following colorectal surgery. Their occurrence is associated with tissue damage, inflammation, angiogenesis, fibrin exudation, and fibrinolysis suppression. Intra-abdominal adhesions are one of the leading causes of severe or even fatal sequelae, such as bowel obstruction, chronic abdominal pain, difficulty in reoperation, and infertility.^1–5^ The reoperation rate associated with intra-abdominal adhesions has been reported as up to 30% to 40%.^6,7^

Antiadhesion agents, such as sodium hyaluronate, fibrin sheets, dextran, and chitosan, have been used to prevent postoperative intra-abdominal adhesion in clinical practice. Among them, sodium hyaluronate gel and chitosan (carboxymethyl chitin) have been shown to be effective in animal studies.^8–11^ There are several mature products of sodium hyaluronate, such as SprayGel^12^ and Seprafilm.^13^ Efficacy in preventing peritoneal adhesions^12,13^ or intra-abdominal adhesions^14,15^ of these products has been demonstrated by some studies, although as indicated in several, their application around anastomosis also increased incidence of anastomotic leakage.^16^ Chitosan has been shown to be effective in preventing adhesion in several animal studies.^9,11,17,18^ Clinical trials are needed to further confirm its efficacy in human.

A temporary enterostomy is usually performed to protect a distal anastomosis from postoperative anastomotic complications after colorectal surgery. Separation of peristomal adhesions under limited exposure from parastomal incision complicates closure of enterostomy. The closure of the stomas, however, provides an opportunity to visually assess peristomal adhesions. The objective of this prospective randomized controlled study was to evaluate the safety and efficacy of sodium hyaluronate gel and chitosan in preventing peristomal adhesions after defunctioning enterostomy.

## METHODS

### Study Design and Patients

This was a prospective blinded randomized controlled study. Patients undergoing colorectal surgery with a defunctioning ileostomy or colostomy at the Department of Colorectal Surgery, the Sixth Affiliated Hospital of Sun Yat-sen University from June 2011 to January 2015 were prospectively enrolled. All patients were >18 years old and underwent defunctioning ileostomy or colostomy with or without bowel resection, followed by a stoma closure procedure at a second stage. Exclusion criteria are as follows: (1) patients with cicatricial diathesis or allergic constitution; (2) patients undergoing a permanent colostomy; (3) patients with a history of operation or adhesion at the peristomal area; (4) patients with a preoperative diagnosis of endometriosis or diffuse peritonitis; (5) use of corticosteroid, immunosuppressant, or receiving peritoneal dialysis within 3 years before the surgery; (6) female patients who were pregnant. The Hospital Ethic Review Board approved the study and all participants provided written informed consent.

### Randomization and Treatment Groups

Participants were randomly assigned to 3 study groups: sodium hyaluronate gel group (SHG group), chitosan group (CH group), or control group (CON group). Randomization number was generated by the computer and kept in a sealed envelope. Patients and surgeons were blinded to the treatment assignment before the surgery. The envelope was unsealed at the operation room before the closure of the abdominal incision and the creation of the enterostomy, to reveal the assignment. For patients randomized to the SHG group or the CH group, sodium hyaluronate gel (Haohai Biological Technology Co, Ltd, Shanghai, China) or chitosan (water-soluble medical chitosan, Qisheng Biological Preparation Co, Ltd. Shanghai, China) was smeared to both limbs of enterostomy. Patients in the control group had standard abdominal procedures without using sodium hyaluronate gel, chitosan, or any other antiadhesion agents.

Three to six months after the enterostomy, the patients underwent second surgery to close the enterostomy. Before the closure of stoma, radiograph was taken to evaluate the integrity of the colorectal anastomosis. Enterostomies were closed with hand-sewn or stapled anastomosis. During the second surgery, the presence and severity of adhesions to the enterostomy site were evaluated by the operating surgeons, who were unaware of the patients’ treatment groups and were different from the ones who performed the initial surgery. All patients received standard care and were kept blinded to their treatment assignments during the study. Follow-up evaluations were performed on the 2nd or 3rd day, 7th day, 30th day, and 12 to 24 weeks postoperation.

### Safety Evaluation

All efficacy and safety outcomes were adjudicated by surgeons who were blinded to patients’ treatment assignment. All participants were included in the safety evaluation. Toxicities to liver and kidney related to the use of sodium hyaluronate gel or chitosan were evaluated within 7 days after enterostomy. Postoperative short-term complications included peristomal cutaneous infection, intra-abdominal hemorrhage, and abscess. Stoma-related complications included stoma stenosis, parastomal hernia, stoma prolapse, and stoma retraction. Long-term postoperative complications included intestinal obstruction and anastomotic leakage. Cancer progression was defined as local recurrence and metastasis.

### Efficacy Evaluation

Patients who received the closure of the enterostomy were included in the efficacy analyses. The primary outcome was the incidence of adhesions present during the follow-up surgery (3–6 months after the initial surgery), defined as 1 or more adhesions to the enterostomy wound or between intestinal tracts. The secondary outcome was the severity of adhesion scored as: 0 = no adhesion; 1 = filmy or negligible, avascular adhesions, separated easily with blunt dissection; 2 = firm or moderately thick, limited vascular adhesions, separated with blunt or sharp dissection; and 3 = fibrotic, dense, well-vascular adhesions, separated only with sharp dissection.^19,20^

### Statistical Analysis

Sample size was calculated using SAS software (version 9.4, SAS Inc, Cary, NC). A sample size of 30 per group was required to detect a difference of 50% in incidence of adhesions, with 80% power (β=0.20) and 5% 2-tailed significance level (α=0.05). A total of 111 patients (37 patients per group) were enrolled in this study, allowing a dropout rate of 20%.

Data were analyzed on an intention-to-treat basis. All analyses were performed using SPSS software (Version 19.0, SPSS, Inc, Chicago, IL). Data were presented as mean ± standard deviation for normally distributed continuous data, median with range for non-normally distributed continuous data, and frequency and percentage for categorical data. One-way analysis of variance (for normally distributed data) and Kruskal–Wallis test (for non-normally distributed data) were used to compare continuous variables among the 3 groups. Between-group differences in categorical variables were analyzed by the Chi-square test or Fisher's exact test. Categorical variables were tested by Kruskal–Wallis analysis. When overall between-group difference was statistically significant, a Nemenyi test was used for further multiple comparisons. All statistical tests were 2-tailed with *P* < 0.05 as statistically significant.

## RESULTS

### Baseline Characteristics

**FIGURE 1:**
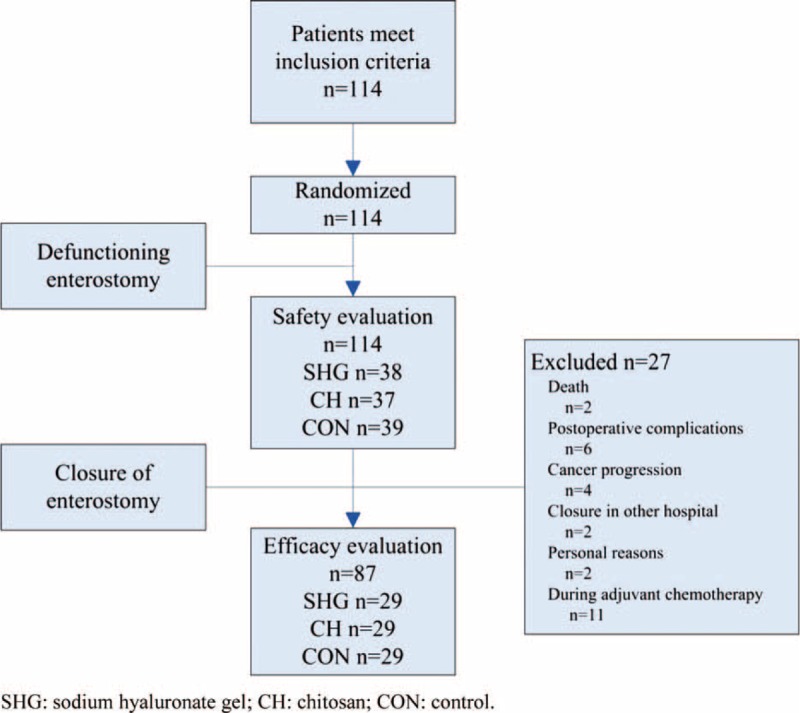
The flowchart of study enrollment.

A total of 114 patients were enrolled and were randomly assigned to 3 groups (Fig. 1). Eighty-seven (76.3%) patients (29 patients in each group) underwent the closure of the enterostomy. Twenty-seven cases did not receive the closure of enterostomy because of death (n = 2, 1.8%, 1 in the SHG group due to tumor metastasis, 1 in the CON group due to cardiovascular accident), postoperative complications (n = 6, 5.3%), cancer progression (n = 4, 3.5%), closure in other hospitals (n = 2, 1.8%), personal reasons (n = 2, 1.8%), or in adjuvant chemotherapy (n = 11, 9.6%).

Table 1 shows the baseline characteristics of the 114 patients included in this study. Age (*P* = 0.74) and gender (*P* = 0.85) did not differ significantly among the 3 groups. The majority of the patients (83.3%) had an underlying disease of colorectal cancer (89.5%, 86.5%, and 74.4% for SHG, CH, and CON groups, respectively, *P* = 0.17). The proportion of patients having preoperative chemotherapy or radiotherapy was similar among the 3 groups (*P* = 0.36 and *P* = 0.70, respectively). No patient received steroids or immunosuppressant within 3 years before the enterostomy.

**TABLE 1:**
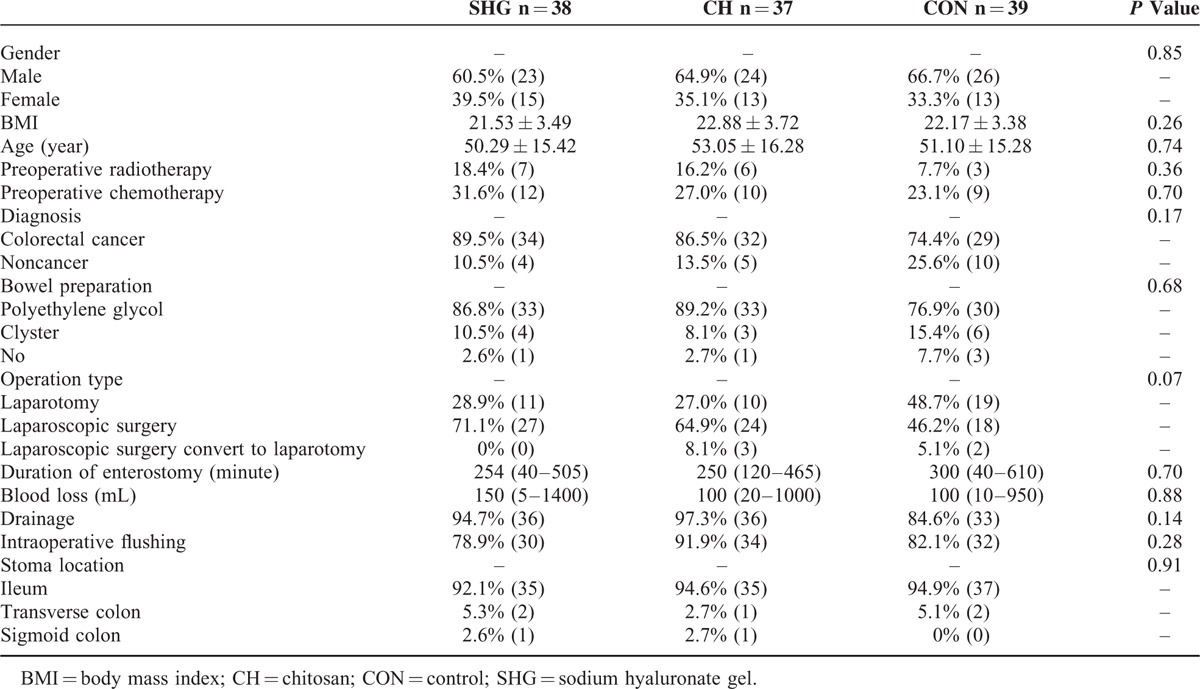
Baseline Characteristics of All Patients Included

Table 2 shows the baseline characteristics of 87 patients (29 patients per group) who received the closure of enterostomy. The mean ± SD age was 50.31 ± 15.61 years (SHG group), 53.0 ± 15.79 years (CH group), and 51.10 ± 14.30 years (CON group), with no significant between-group difference (*P* = 0.79). There was a slight male preponderance in the CON group, but the difference was not significant (*P* = 0.21). Patients were comparable among groups in terms of clinical characteristics. Eighty-three percent of patients had colorectal cancer (89.7% in the SHG group, 86.2% in the CH group, and 72.4% in the CON group, *P* = 0.18). Preoperative chemotherapy and radiotherapy were similar among the 3 groups (*P* = 0.61 and *P* = 0.84, respectively).

**TABLE 2:**
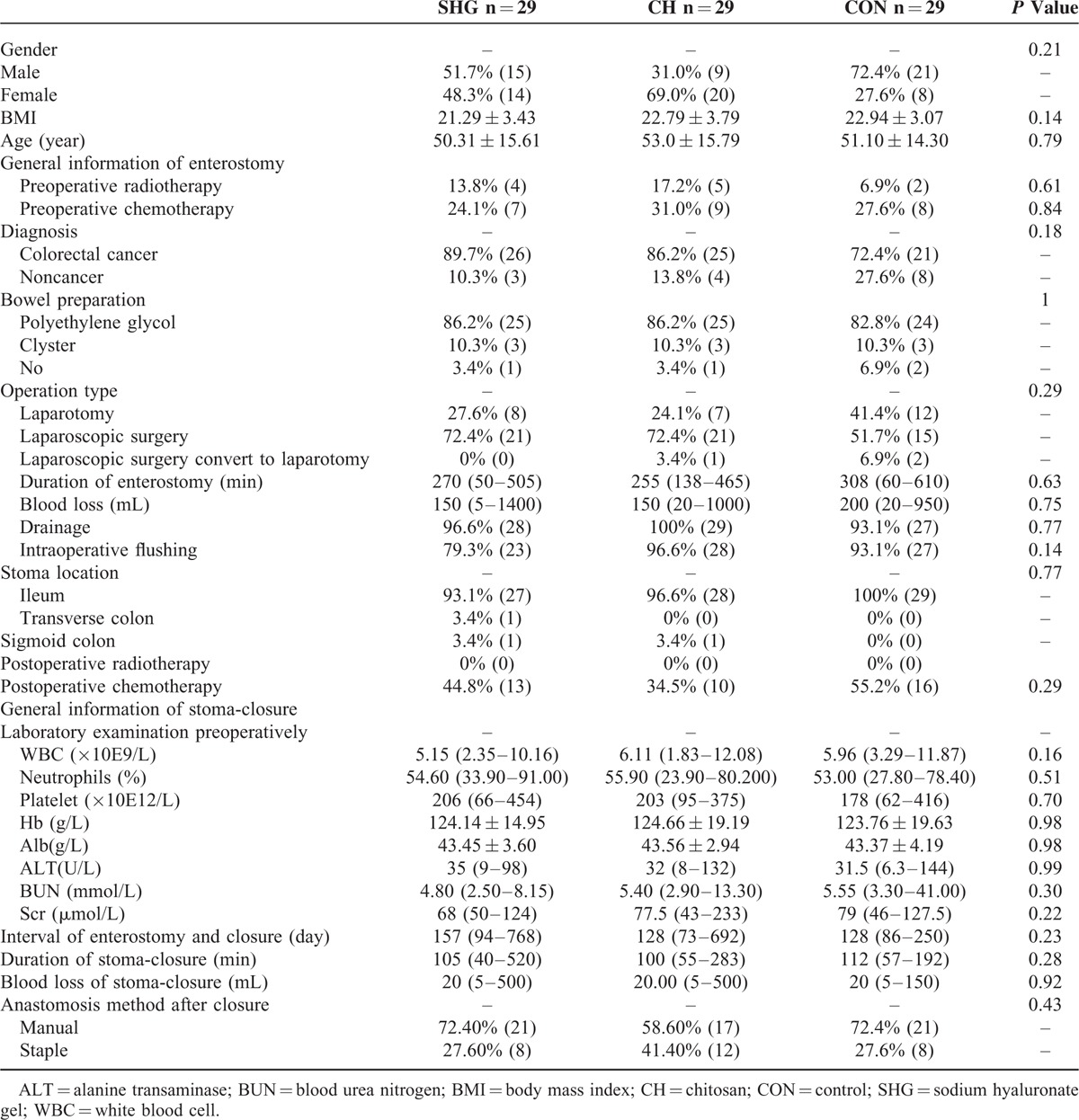
Baseline Characteristics of Patients Received Closure of Enterostomy

### Safety Outcomes

All 114 patients were included for safety evaluation and results are shown in Table 3. Median follow-up duration was 22.77 months (SHG group), 15.57 months (CH group), and 28.07 months (CON group), with no between-group difference (*P* = 0.55). Duration of hospitalization after enterostomy did not differ among the 3 groups (median duration: 12.5 vs 10 vs 12, *P* = 0.15). There was no significant toxicity to liver or kidney related to the use of sodium hyaluronate gel or chitosan. Liver function, renal function, and white blood cell (WBC) levels within 2 weeks after the initial surgery were comparable among the 3 groups (all *P* > 0.05).

**TABLE 3:**
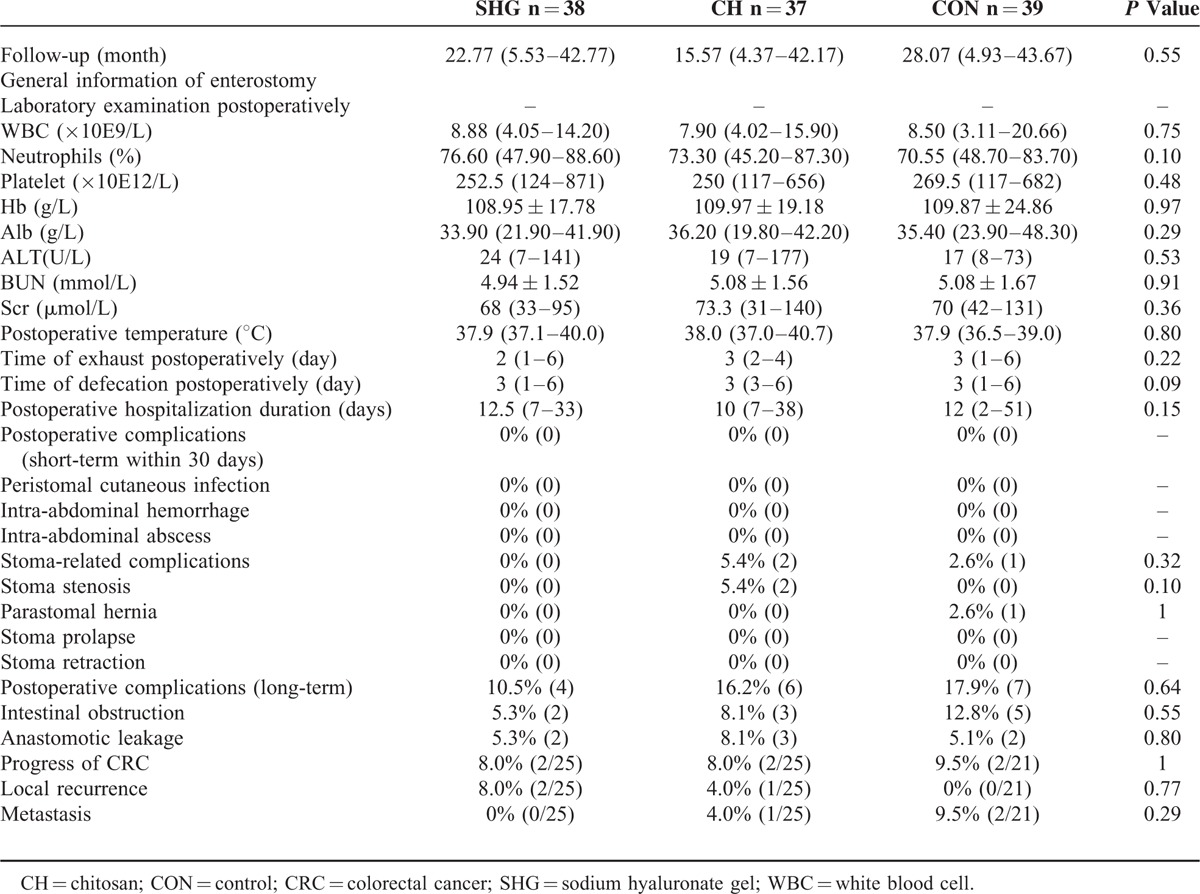
Safety Outcomes

Incidence of stoma-related complications was comparable among groups (Table 3). No patient developed stoma prolapse or retraction. Two patients in the CH group developed stoma stenosis and 1 in the CON group developed parastomal hernia, with no significant between-group difference (all *P* > 0.05). No patient developed short-term postoperative complication such as peristomal cutaneous infection, intra-abdominal hemorrhage, or abscess. Incidence of long-term postoperative complication was similar among groups. Anastomotic leakage occurred in 2 patients in the SHG group, 3 in the CH group, and 2 in the CON group, with no significant between-group difference (*P* = 0.77). Two patients in each group developed cancer recurrence or metastasis after surgery, with no significant between-group difference. Other safety outcomes, including interval between enterostomy and closure, operation duration of stoma-closure, and blood loss during the operation, were also comparable among the 3 groups (Table 3, all *P* > 0.05).

### Efficacy Outcomes

Eighty-seven patients (29 patients per group) who received the closure of enterostomy were included in the efficacy evaluation (Table 4). Incidence of peristomal adhesion in the SHG group (62.1%) and the CH group (62.1%) were lower than that in the CON group (82.8%), but the difference was not significant (*P* = 0.15). Significant between-group difference was found in the adhesion score (*P* = 0.02). Post-hoc analyses showed significantly higher adhesion score in the CON group than that in the CH group (*P* = 0.03) and difference between the CON group and the SHG group was marginally significant (*P* = 0.05). Patients in SHG and CH groups were less likely to develop dense adhesion than those in the CON group (31.0% vs 27.6% vs 62.1%, *P* = 0.01). Post-hoc analyses showed marginally significant difference between the SHG group and the CON group (*P* = 0.05) and significant difference between the CH group and the CON group (*P* = 0.01).

**TABLE 4:**
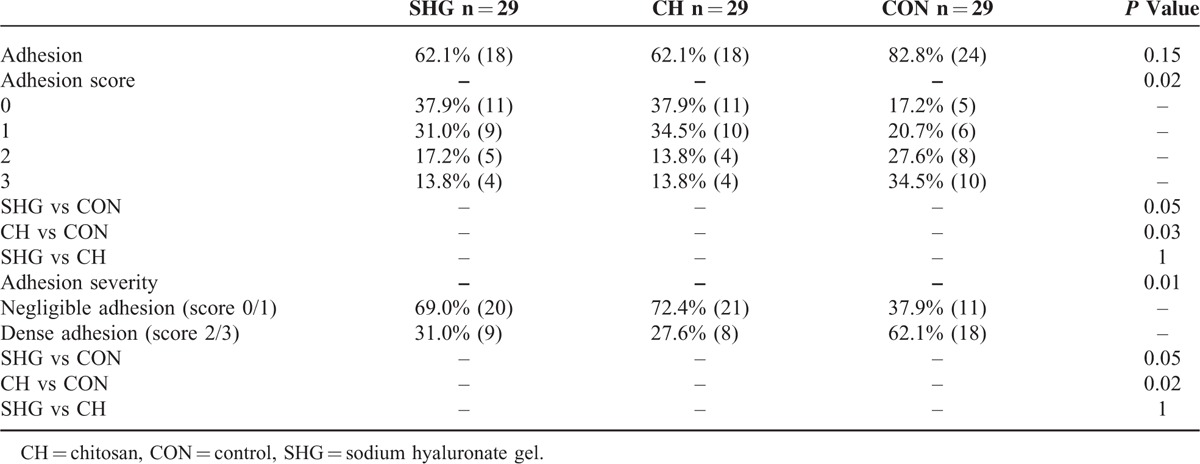
Efficacy Outcomes

## DISCUSSION

Intra-abdominal adhesions arise after up to 94% abdominal or pelvic surgery^21,22^ and are an importance source of common and even fatal postoperative complications, such as intestinal obstruction, chronic abdominal pain, higher complication rates in subsequent operations, and infertility.^1–5^ Reoperation rate due to intra-abdominal adhesions is up to 30% to 40% of patients.^6,7^ Meticulous surgical techniques^4,5^ and laparoscopic surgery^23^ could prevent, to some extent, intra-abdominal adhesions. However, small bowel obstruction can still occur in 7% to 8% patients after laparoscopic colorectal surgery.^24,25^

Fibrin deposition and matrix formation start within 12 h after damage of peritoneal and serosa tissue, which is a major contributing factor in the formation of adhesions.^26^ Normal fibrinolytic activity of tissue is further compromised under ischemic conditions during surgical procedures, allowing fibrin matrix to persist and mature into an organized fibrous adhesion in ∼5 days.^26^ A new mesothelium normally forms within 7 to 9 days.^24^ Sodium hyaluronate gel and chitosan reduce adhesion formation by separating surfaces of surgically damaged tissues. In addition, chitosan has an antibacterial effect and can inhibit the proliferation of fibroblasts.^9^

There are several studies evaluating the efficacy of sodium hyaluronate in the prevention of intra-abdominal adhesions. The majority of these studies used membranous sodium hyaluronate,^27,28^ and the others used liquid forms. Several studies evaluated the efficacy of sodium hyaluronate in the prevention of intra-abdominal adhesions during closure of enterostomy. Conflicting results on whether this agent could prevent intra-abdominal adhesions have been reported in these studies.^29,30^ Chitosan has been shown to be effective in animal studies, although evidence of its efficacy in human in colorectal procedures is limited. Our study used gelatinous sodium hyaluronate and chitosan, which stay in the intestinal tracts around the stoma after being smeared. Our results showed a comparable safety profile of these 2 antiadhesion agents and that incidence of adhesions appeared to be lower with these agents, albeit not statistically significant. Compared with the control group without any antiadhesion treatment, occurrence of dense adhesions was significantly reduced with these 2 agents.

Our study also investigated whether these antiadhesion agents would induce toxic reactions or increase the risk of other complications.^31^ We evaluated WBC levels and liver and renal function 1 to 2 weeks after the initial surgery and did not find significant toxic event associated with these agents. No stoma prolapse or retraction occurred in this study and other safety outcomes, such as stoma-related complications and tumor progression, were comparable to those in the control group. Previous studies reported that membranous sodium hyaluronate increased the incidence of anastomotic leakage.^16^ Our results, however, showed that risk of anastomotic leakage did not increase with either agent. That may be explained by the fact that application of gelatinous adhesion barriers is simple and straightforward, requiring minimal training, and they stay in the intestinal tracts around the stoma after being smeared without affecting the anastomosis. Nonetheless, cautions are required when applying these agents at the anastomosis area.

This prospective randomized controlled study is the first study to investigate the antiadhesion effect of chitosan in human. Both formation and severity of intestinal adhesions around stoma were visually assessed in this study during the closure of enterostomy. The study has several limitations. First, sample size was small and follow-up duration was relatively short. Conclusions on long-term efficacy and safety of sodium hyaluronate gel and chitosan in the prevention of adhesions cannot be drawn. Second, although the study was evaluator-blinded, adhesion is difficult to quantify and the scoring of adhesion was based on a subjective evaluation. Third, some postoperative complications, such as pelvic pain, fertility outcomes, and quality of life, were not assessed.

In conclusion, in this randomized controlled study, we evaluated the safety and efficacy of 2 antiadhesion agents, sodium hyaluronate gel and chitosan, compared with control group without antiadhesion treatment. Occurrence and severity of intra-abdominal adhesions were assessed during closure of enterostomy. Our results showed a comparable safety profile of these 2 antiadhesion agents and that incidence of adhesions appeared to be lower with these agents, albeit not statistically significant. Compared with control group without any antiadhesion treatment, the severity of postoperative adhesion was significantly decreased in sodium hyaluronate gel group and chitosan group.
